# Metabolite profiling of abalone (*Haliotis iris*) energy metabolism: a Chatham Islands case study

**DOI:** 10.1007/s11306-022-01907-6

**Published:** 2022-07-13

**Authors:** Leonie Venter, Andrea C. Alfaro, Thao Van Nguyen, Jeremie Zander Lindeque

**Affiliations:** 1 Aquaculture Biotechnology Research Group, Department of Environmental Science, School of Science, Auckland University of Technology, Private Bag 92006, Auckland, 1142 New Zealand; 2 NTT Hi-Tech Institute, Nguyen Tat Thanh University, Ho Chi Minh City, Vietnam; 3 Human Metabolomics, North West University, Potchefstroom Campus, Private Bag X 6001, Potchefstroom, 2520 South Africa

**Keywords:** Abalone, Anabolism, Chatham Islands, Metabolomics, Populations

## Abstract

**Introduction:**

The Chatham Islands has some of the most prized black-footed abalone (*Haliotis iris*) beds in New Zealand. This well-managed fishery includes restrictions on catch and size limits, selective fishing methods, and shellfish management. However, recent declines in biomass and growth parameters have prompted omics research to characterise the biological responses of abalone, potentially contributing towards animal management strategies.

**Objectives:**

The aim of this study was to characterise the metabolite profiles of slow and fast growing, juvenile and adult abalone, relating to metabolites supporting energy metabolism.

**Methods:**

A gas chromatography–mass spectrometry metabolite profiling, applying methyl chloroformate alkylation, was performed on juvenile and adult abalone samples collected from Point Durham and Wharekauri sites, Chatham Islands, New Zealand.

**Results:**

The results obtained from haemolymph and muscle samples indicated that abalone from the fast-growing area, Wharekauri, fuelled metabolic functions via carbohydrate sources, providing energy for fatty acid and amino acid synthesis. Conversely, higher amino acid levels were largely utilised to promote growth in this population. The metabolism of juvenile abalone favoured anabolism, where metabolites were diverted from glycolysis and the tricarboxylic acid cycle, and used for the production of nucleotides, amino acids and fatty acids.

**Conclusions:**

This research provides unique physiological insights towards abalone populations supporting the use of metabolomics as a tool to investigate metabolic processes related to growth. This work sets the stage for future work aimed at developing biomarkers for growth and health monitoring to support a growing and more sustainably abalone fishery.

**Graphical abstract:**

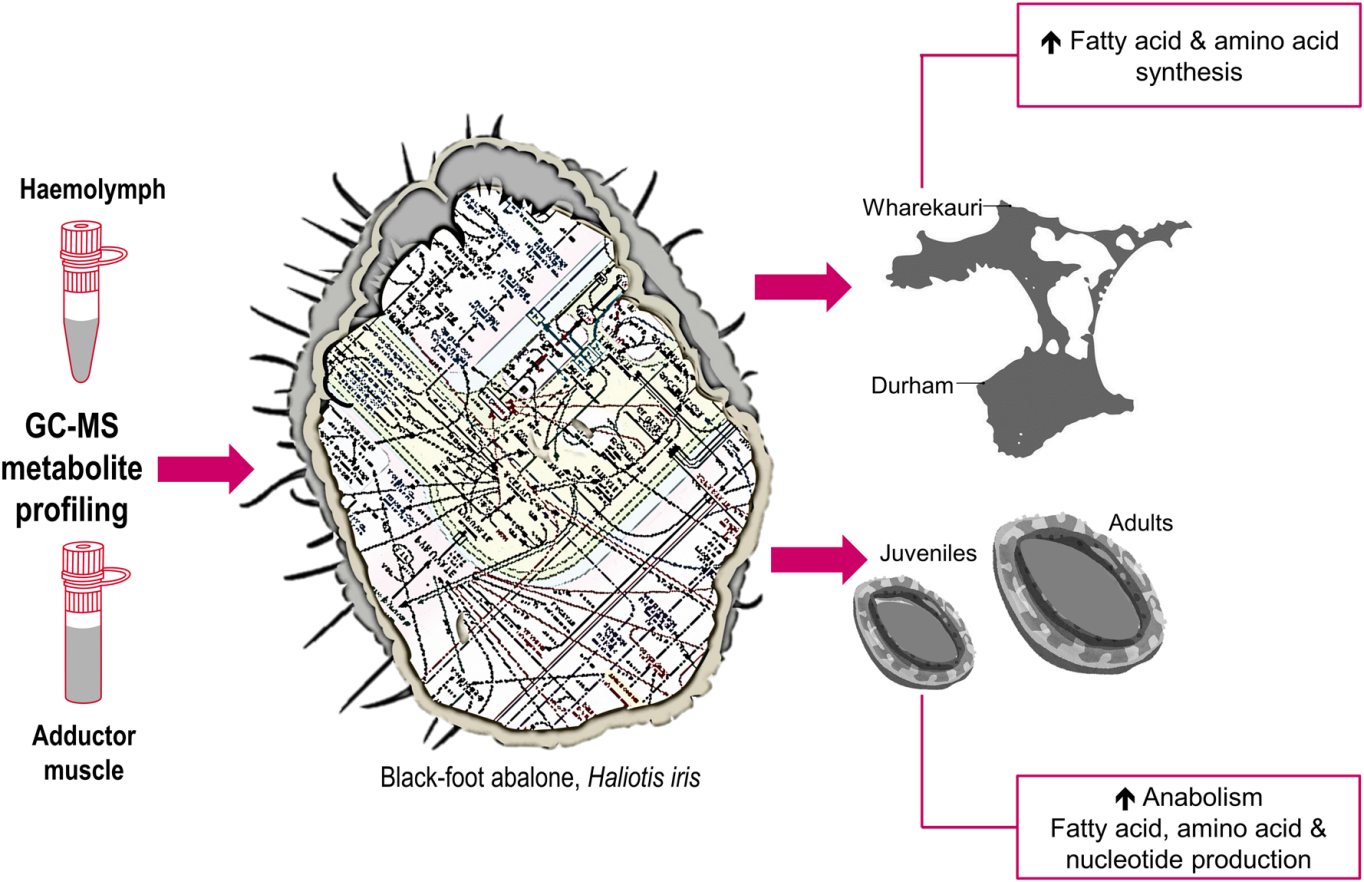

**Supplementary Information:**

The online version contains supplementary material available at 10.1007/s11306-022-01907-6.

## Introduction

The New Zealand black-footed abalone (*Haliotis iris*) is endemic to the coastal waters of New Zealand, encompassing cultural, ecological and economic importance (Will et al., 2015). In 2019, abalone exports were worth $50 million, including shell, by-products and nutraceutical sales. The commercial abalone fishery is managed in accordance with total allowable commercial catch systems. Of the managed areas, the Chatham Islands contribute more than a quarter to the national 720 tonnes of commercially harvested wild abalone (PMCSA, 2021). In recent years, a decline in abalone biomass has been observed in the Chatham Islands, resulting in new research and managing approaches to ensure the fishery is sustainable for future generations. Translocation and reseeding programmes are examples of such initiatives, where new spawning banks are established, or where abalone with slower growth are moved to areas with higher recorded growth rates (Fisheries`New`Zealand, 2019a). Furthermore, the implementation of a minimum harvest size of 125 mm helps to increase the spawning biomass and allows areas with abundant abalone growth, previously depleted, to recover (Fisheries`New`Zealand, 2019b).

Characteristically juvenile abalone prefer habitats sheltered from wave exposure, but when they transition to adults, they are often found in areas of high wave exposure and dense macroalgal cover (Shaffer & Rovellini, 2020). As abalone of different life stages have different habitat preferences, investigations into abalone performance need to be size- and habitat-specific to assist management decisions. In addition, environmental factors, such as food availability also affect abalone growth and health and need to be considered for physiological assessments (Nguyen et al., 2021). To fill gaps in our knowledge regarding *H. iris* growth and performance, insights into abalone physiology and the effects of various biotic and abiotic factors on the organism’s growth and health are essential (Venter et al., 2018a). This information is also crucial to improve our understanding of the physiological mechanisms that underpin abalone population variations, susceptibility and resilience to environmental change to ensure a sustainable future for wild stocks and the fishery.

Various tools and techniques exist to measure physiological parameters of molluscs. These are often focused at biological responses at the molecular, cellular, biochemical, physiological, or behavioural level (Waller & Cope, 2019), typically measured using omics approaches (Nguyen et al., 2021). Generally, omics-based approaches identify sets of gene products such as transcripts, proteins and metabolites, within a biological sample (rather than single products at a time) (Alfaro & Young, 2018). Of these, metabolomics is at the endpoint of the omics cascade, and the closest to the cell’s functional phenotype, which is dictated by both the genome and environment, making metabolites more likely to contribute to the functional state of cells and serve as a direct signature of biochemical activity (Venter et al., 2018a).

The aim of this research was to characterise the metabolite profiles of juvenile and adult abalone collected from two sites at the Chatham Islands with different animal growth rates. Specifically, we study the endo- and exometabolome of abalone adductor muscle and haemolymph samples, respectively, using a gas chromatography-mass spectrometry (GC–MS) metabolomics approach, and profile metabolites contributing to energy metabolism adding new knowledge to a larger project investigating *H. iris* physiology.

## Materials and methods

### Abalone sampling

Abalone were collected under special permit (720, client number 9791209) issued by Fisheries New Zealand, from two catch zones (as defined by the Ministry of Fisheries statistical area maps) around the Chatham Islands: 1) Zone 437—Awatotara Creek- Awa Raku (locally referred to as Point Durham: Lat -44.009608, Long -176.687622); 2) Zone 415—Te Awamuti—Okahu Creek (locally referred to as Wharekauri: Lat -43.705298, Long -176.584711). Point Durham is classified as a low recovery area, which is not fished often due to the slow growth of abalone in this area, while Wharekauri is classified as a high fished (abalone fast growth) area.

Juvenile (n = 20) and adult (n = 20) abalone were collected by divers from Point Durham and Wharekauri sites. Haemolymph were taken from the pedal sinus and transferred into cryovials followed by snap-frozen using liquid nitrogen. Animals were weighed ± 0.01 g, and the shell lengths and heights were measured ± 0.10 mm. Following shucking, a sample of the adductor muscle was taken from the ventral surface towards the central point, where the muscle attaches to the shell, and snap-frozen. All samples were shipped to the laboratory on dry ice and stored at − 80 °C until metabolomics analyses.

### Metabolomics analysis

Frozen haemolymph (500 µL) and approximately 10 mg of ground muscle sample, together with 20 µL of internal standard (10 mM l-alanine-2,3,3,3-d_4_) were dried and extracted using a two-step methanol:water solution. Extracted metabolites were derivatised by methyl chloroformate (MCF) alkylation and analysed via GC–MS (Alfaro et al., 2021).

Quality control (QC) samples were included in every batch by preparing a pooled mixture of haemolymph or muscle tissue (Broadhurst et al., 2018). Additionally, a derivatised sample blank containing the internal standard, an in-house prepared derivatised amino acid mix and a non-derivatised alkane mix were also injected and analysed for QC purposes (Young et al., 2019).

The MCF derivatives were analysed with an Agilent GC7890B and autosampler coupled to a MSD5977A, with a quadrupole mass selective detector (EI) operated at 70 eV, using a ZB-1701 GC capillary column (30 m × 250 μm id × 0.15 μm with 5 m stationary phase) and helium as carrier gas (flow of 1 mL min^−1^). Samples (1 μL) were injected under pulsed splitless mode with the injector temperature at 260 °C, following a temperature program as reported by Alfaro et al. (2021). Identification of compounds was carried out using mass spectra acquired in scan mode from 38 to 550 amu, with detection threshold of 100 ion counts (view supplementary material for detailed description of methods used) (Smart et al., 2010).

### Data processing

Raw spectra were processed using AMDIS (v2.66) software. Metabolite identifications and peak integrations were conducted using Chemstation software and customised R-XCMS based scripts (Aggio et al., 2011), based on an in-house mass spectral library of MCF derivatised commercial standards. Compound identifications were based on matches (≥ 70%) to both the MS spectrum of the derivatised metabolite and its respective retention times. Identified compounds can be assigned a level 1 identification confidence level (Schymanski et al., 2014). Unknown features are not shown. The data matrices of peak intensities were pre-processed for quality control purposes prior to statistical analyses using the web-based tool MetaboAnalyst 5.0 (Pang et al., 2021). Data were normalised to the peak intensity of the internal standard and to sample biomass in the case of muscle tissue (Nguyen et al., 2018).

### Statistical analysis

QC samples analysed amongst the batches were assessed in terms of coefficient of variance percentages and data were evaluated for within batch effects. The data were generalised log (glog) transformed and two-way analysis of variance (ANOVA) was used to determine the influence of collection site and animal life stage (between subject, *p* < 0.05). The metabolite response was further analysed and visualized in a blocked manner, with principal component analysis (PCA) (Xu & Goodacre, 2012), and by constructing individual metabolite maps of metabolite findings linked to animal collection site and life stage. Grouping of experimental groups on the PCA scores plots were highlighted with the use of 95% confidence ellipses around each group. Data analyses for morphological parameters were conducted with SPSS® software, version 23.0, using student t-tests to identify differences between collection site and life stage (*p* < 0.05).

## Results and discussion

**Fig. 1:**
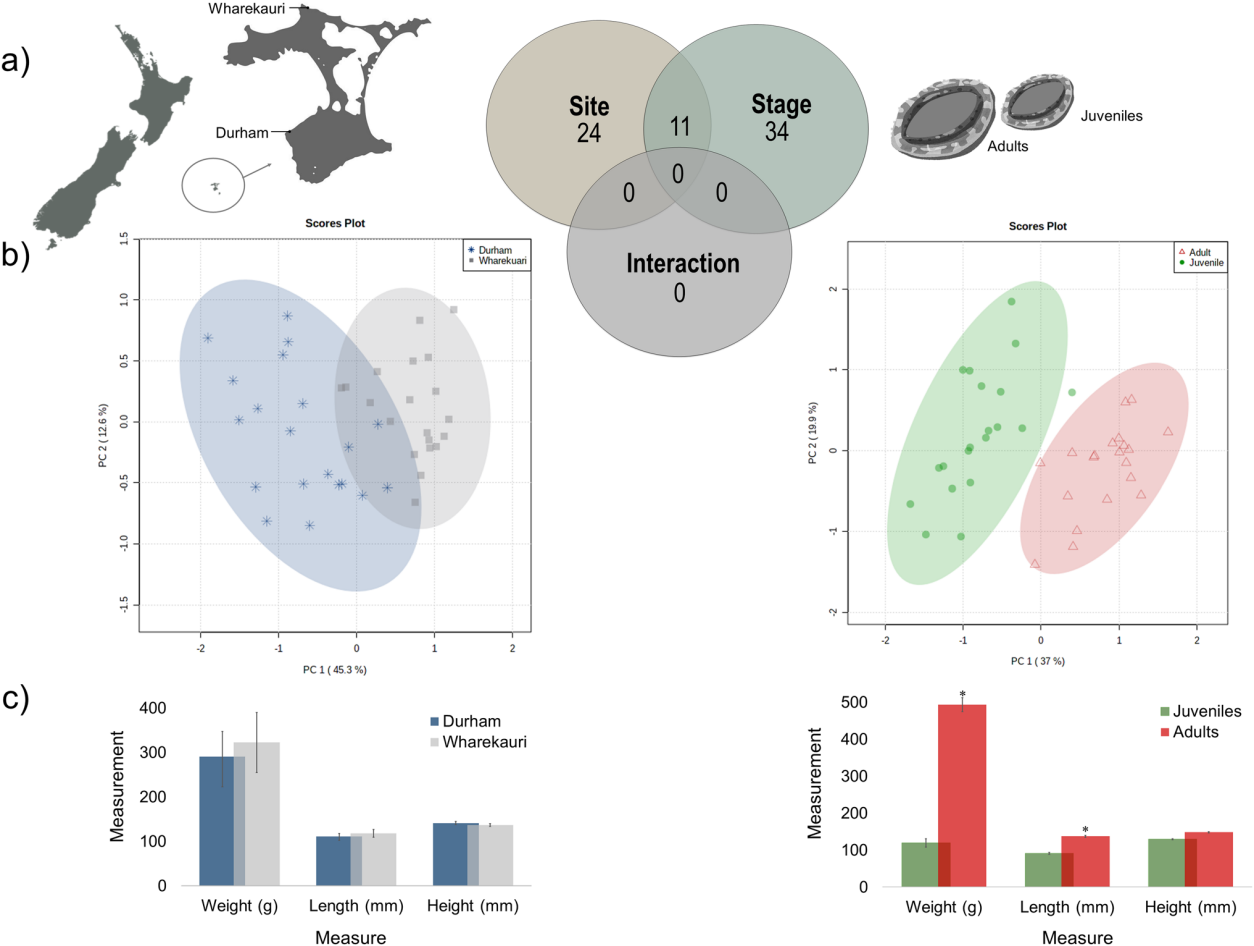
Overview of metabolomics results as a Venn diagram (**a**); PCA plots (**b**) and morphometric (weight, length and height) measures (**c**) from two abalone collection sites (Durham and Wharekauri) and abalone of two life stages (adults and juveniles)

Marine invertebrate populations generally span habitats with a range of physical and biological characteristics (Sebens, 2002), which can significantly influence their metabolism, growth and health. This link between the environment and growth was investigated in this study by focusing on metabolic differences between slow growing abalone, collected from Durham, and fast-growing abalone, collected from Wharekauri (Fig. 1a). Both adult and juvenile abalone were studied in an attempt to reveal key metabolic activity that could explain increased growth in the Wharekauri abalone. Understanding how the environment influences the metabolism and growth of abalone in fished populations is central to effective fisheries management (Naylor et al., 2006). Metabolic studies have previously been performed on juvenile or adult *H. midae* (Venter et al., 2019), *H. iris* (Grandiosa et al., 2018), *H. fulgens* (Tripp-Valdez et al., 2019) and *H. discus hannai* (Xu et al., 2020), yet metabolomics characterisation of a population to guide stock and fisheries management or translocation practises remains an untouched subject.

The morphological parameters (Fig. 1c), showed an average wet weight of 290.55 ± 57; 322.05 ± 68 g, shell length of 110.42 ± 7; 117.60 ± 8 mm and shell height of 140.90 ± 4; 136.70 ± 3 mm (average ± standard error) for abalone collected from Durham and Wharekauri sampling sites, respectively. No differences were found in morphometric measures between animals collected from Durham and Wharekauri. Juvenile abalone had an average wet weight of 119.15 ± 11 g, shell length of 90.91 ± 2; mm and shell height of 129.30 ± 1 mm. While adult abalone had an average wet weight of 493.45 ± 19 g, shell length of 137.11 ± 3; mm and shell height of 148.30 ± 2 mm (average ± standard error). Adult abalone were significantly heavier and longer than juvenile abalone (*p* < 0.05).

Based on two-way ANOVA, the largest number of metabolites were affected by animal life stage, yielding a total of 45 metabolites. Abalone collection site as experimental factor resulted in 35 significant different metabolites, as shown in the Venn diagram (Fig. 1a). The covariance of these metabolites was visualised with PCA in a multiblock manner, to highlight their combined discriminatory power (Xu & Goodacre, 2012) (Fig. 1b). A moderate overlap was seen in the scores of the different collection sites (across life stages), showcasing the lower impact of this experimental factor on the metabolism of adult and juvenile abalone from both sites. The relevant metabolic variation due to collection site is mainly captured by PC1, which explains 45.3% of the variance. PC2 explains 12.6% of the variation in the data, which is mostly unrelated to collection site, adding to a larger variance in the metabolism of the abalone from Durham. Complementary loadings data are provided in the supplementary material (Table S1 and Figure S1). Similarly, the PCA comparison of life stages (from both sites) gave clear distinctions between juvenile and adult abalone, with PC1 of the plot explaining most (37%) of this variance. PC2 explains 19.9% of the variation in the data, largely unrelated to life stage. Complementary loadings data are provided in the supplementary material (Table S2 and Figure S2).

**Fig. 2:**
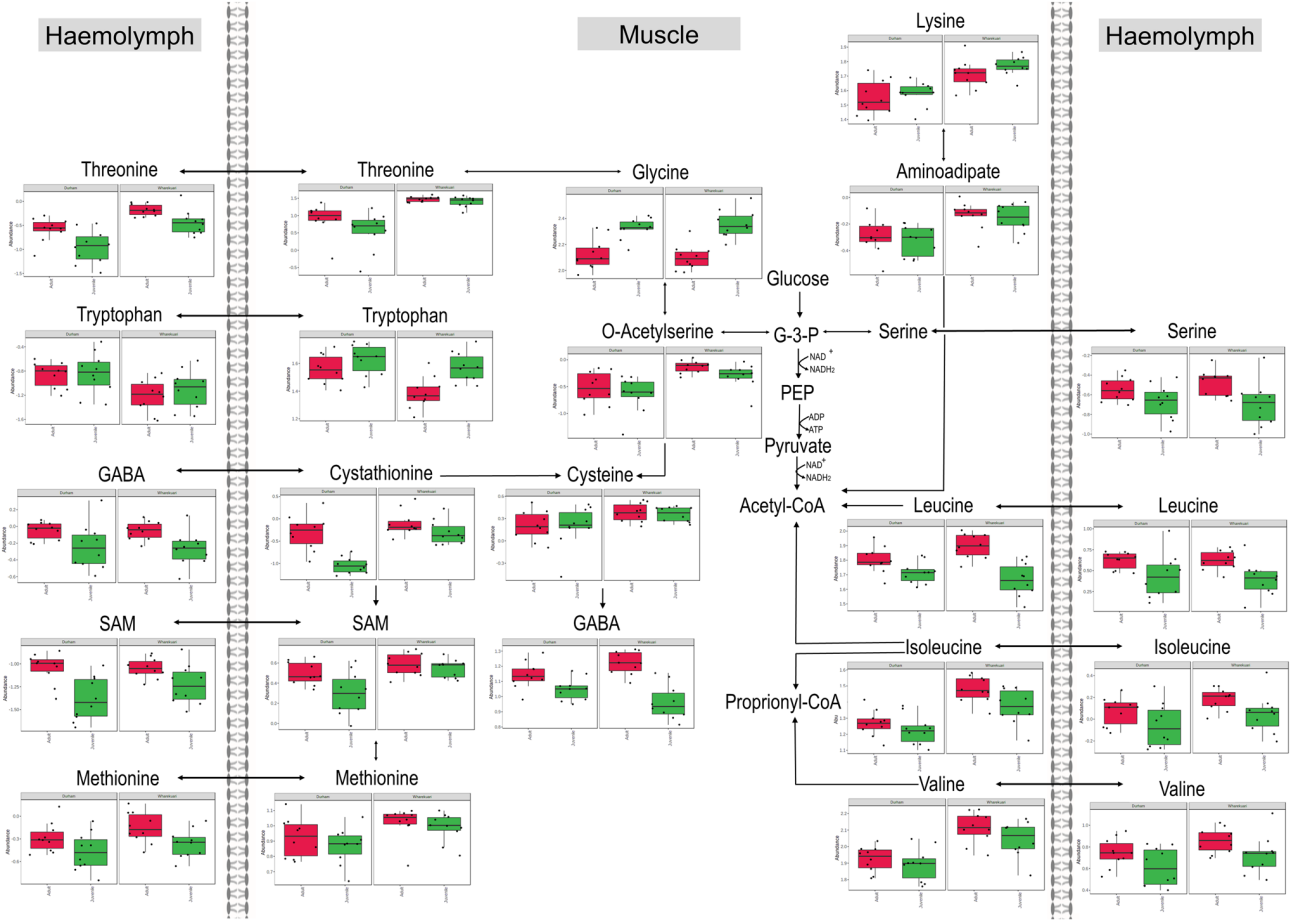
Central carbon metabolism highlighted in *H. iris* haemolymph and muscle tissues. Metabolite abundance of adult abalone are represented by the red plots and juvenile abalone by the green plots. The Durham collection site is shown by the left set of plots and the Wharekauri site by the right set

**Fig. 3:**
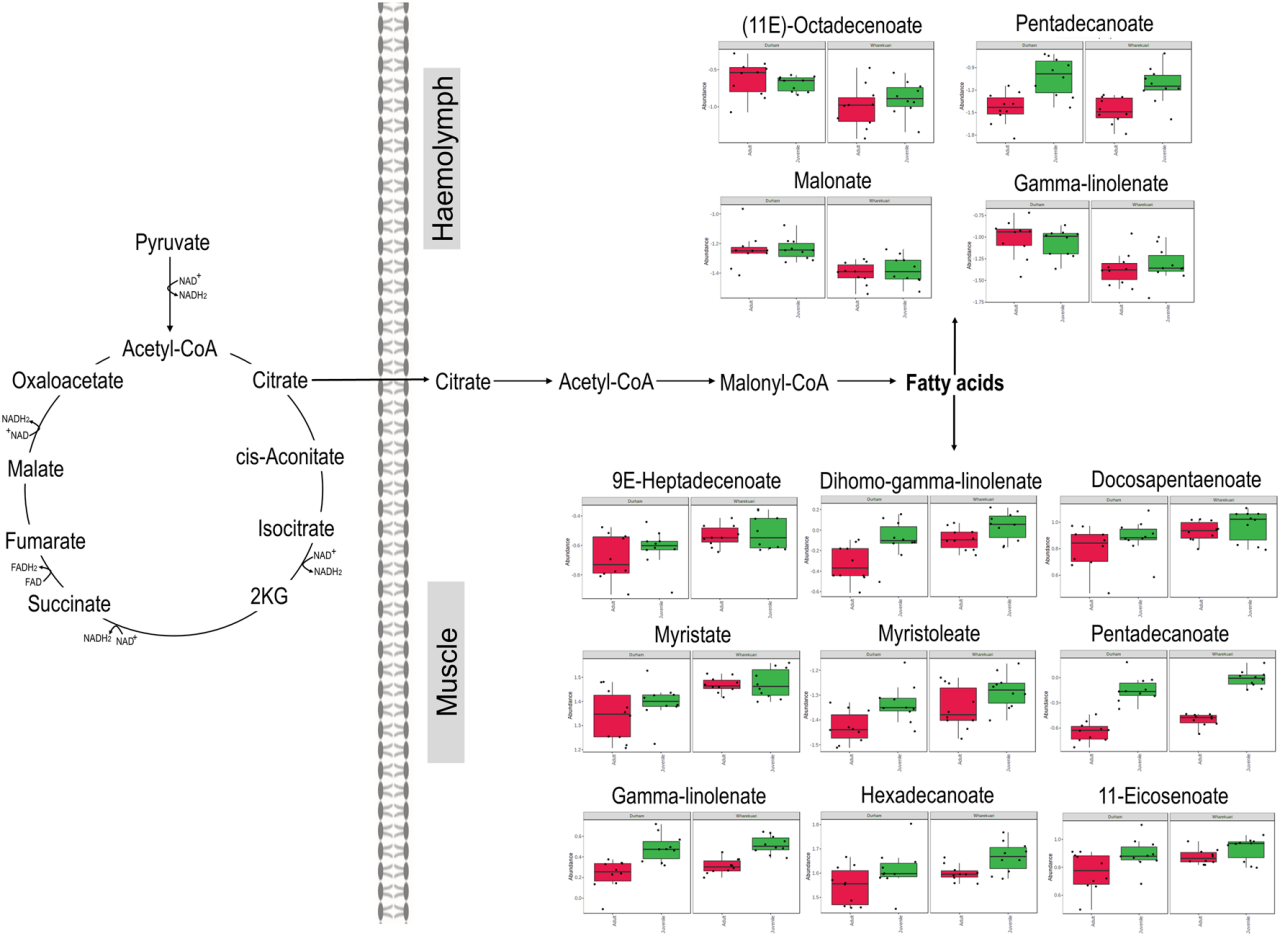
Metabolite responses of *H. iris* haemolymph and muscle tissues linked to lipogenesis (metabolite abundance shown as red = adults, green = juveniles, left set = Durham, right set = Wharekauri)

**Fig. 4:**
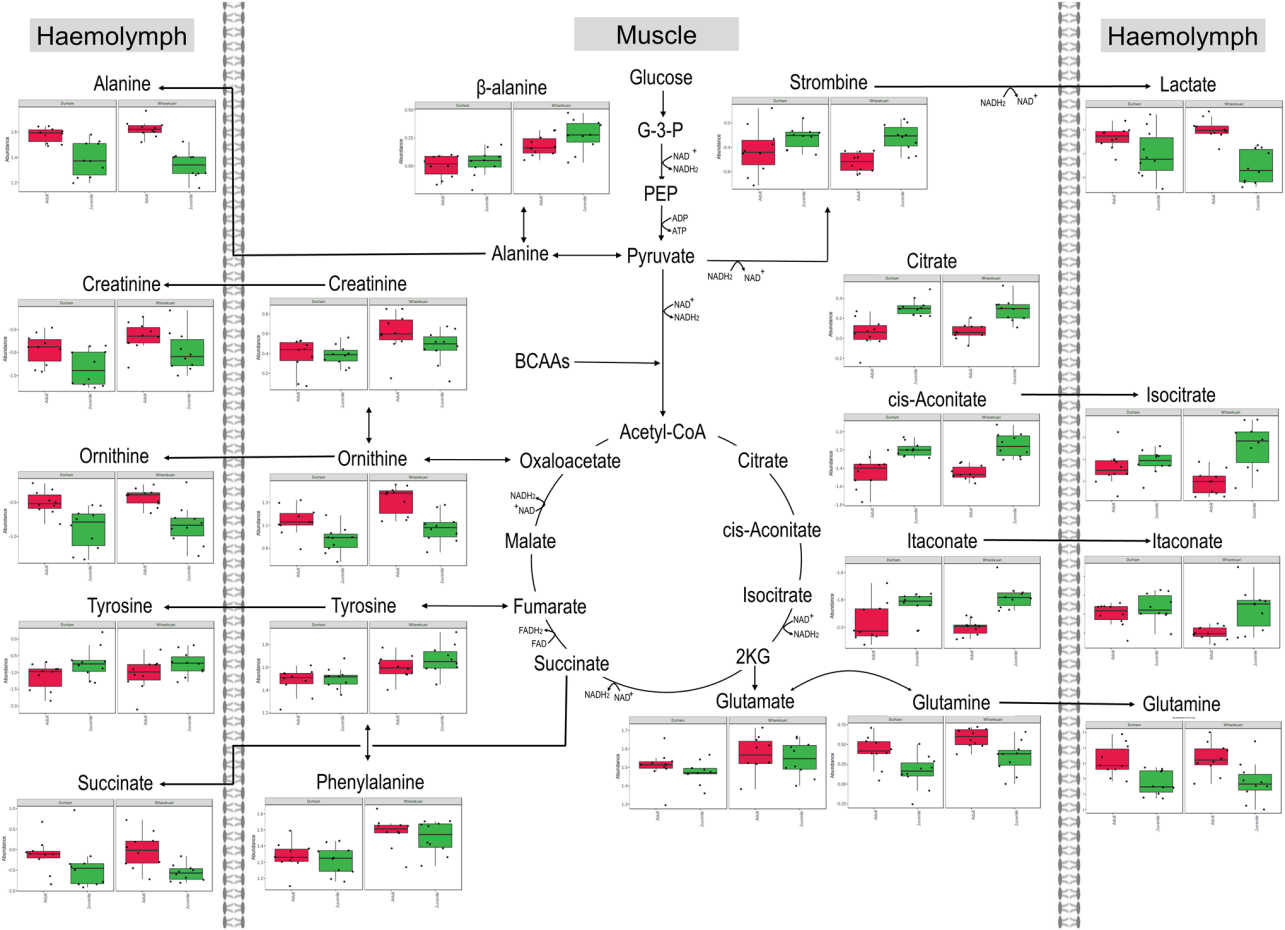
Amino acid metabolite responses of *H. iris* haemolymph and muscle tissues (metabolite abundance shown as red = adults, green = juveniles, left set = Durham, right set = Wharekauri)

The metabolites that differed significantly between collection sites are shown in Table 1 as metabolite response of Wharekauri (compared to Durham) abalone (*p* < 0.05). Table 2 shows the metabolites that differed significantly in the juvenile abalone when compared to the adults, irrespective of the collection site. Metabolites in bold in Tables 1 and 2 indicate significance within the site or stage groups. A matching online Kyoto Encyclopedia of Genes and Genomes (KEGG) database ID (Kanehisa et al., 2021), metabolite classification, tissue finding, and the metabolite response is found in both tables. These findings are further illustrated in the metabolic maps of Figs. 2, 3 and 4.

**Table 1:** The metabolite levels significantly different in abalone collected at Wharekauri compared to Durham

Metabolite	KEGG Compound ID	Classification	Tissue	Site (*p* < 0.05)	Stage (*p* < 0.05)	Interaction (*p* < 0.05)	Response Wharekuari
(11E)-Octadecenoate	C08367	Fatty acid	Haemolymph	**4.39E−03**	7.62E−01	9.83E−01	↓↓
1-Aminocyclopropane-1-carboxylate (ACC)	C01234	Alpha amino acid	Muscle	**1.94E−05**	1.29E−01	9.74E−01	↑↑↑
2-Oxoglutarate	C00026	Keto acids	Muscle	**1.94E−05**	**6.82E−04**	9.37E−01	↓↓↓
4-Hydroxyphenylacetate	C13636	Phenol ester	Muscle	**1.42E−04**	2.60E−01	9.37E−01	↑↑↑
9E-Heptadecenoate	C16536	Fatty acid	Muscle	**7.60E−03**	3.63E−01	9.37E−01	↑
Aminoadipate	C00956	Alpha amino acid	Muscle	**3.06E−04**	5.83E−01	9.74E−01	↑↑↑
beta-Alanine	C00099	Amino acid	Muscle	**1.94E−05**	2.02E−01	9.37E−01	↑↑↑
cis-Aconitate	C00417	Organic acid	Haemolymph	**1.91E−02**	3.70E−01	9.83E−01	↑
Creatinine	C00791	Alpha amino acid	Muscle	**1.44E−02**	3.13E−01	9.37E−01	↑
Cystathionine	C02291	Amino acid	Muscle	**7.31E−05**	**5.20E−05**	1.55E−01	↑↑↑
Cysteine	C00793	Amino acid	Muscle	**2.09E−02**	8.31E−01	9.74E−01	↑
Dihomo-gamma-linolenate	C03242	Fatty acid	Muscle	**2.63E−03**	**4.32E−03**	9.37E−01	↑↑
Docosapentaenoate (DPA)	C16513	Fatty acid	Muscle	**2.45E−02**	2.12E−01	9.37E−01	↑
gamma-Linolenate	C06426	Fatty acid	Haemolymph	**4.50E−04**	9.57E−01	9.83E−01	↓↓↓
Glutamate	C00025	Amino acid	Muscle	**3.97E−02**	4.16E−01	9.74E−01	↑
Glutamine	C00064	Amino acid	Muscle	**1.58E−02**	**6.66E−04**	9.74E−01	↑
Histidine	C00135	Amino acid	Muscle	**1.22E−03**	8.31E−01	9.74E−01	↑↑
Isoleucine	C00407	Amino acid	Muscle	**1.59E−05**	**3.97E−02**	9.37E−01	↑↑↑
Lysine	C00739	Amino acid	Muscle	**1.80E−05**	2.73E−01	9.37E−01	↑↑↑
Malonate	C00383	Dicarboxylic acid	Haemolymph	**3.27E−04**	6.61E−01	9.83E−01	↓↓↓
Methionine	C00073	Amino acid	Muscle	**1.18E−02**	3.47E−01	9.37E−01	↑
Myristate (C14)	C06424	Fatty acid	Muscle	**4.74E−04**	3.13E−01	9.37E−01	↑↑↑
Myristoleate	C08322	Fatty acid	Muscle	**2.80E−02**	**5.18E−03**	9.37E−01	↑
O-Acetylserine	C00979	Carboxylic acid	Muscle	**4.52E−04**	2.05E−01	9.37E−01	↑↑↑
Ornithine	C00077	Amino acid	Muscle	**2.90E−03**	**4.89E−05**	9.37E−01	↑↑
Palmitolate (C16:1n9)	C08362	Fatty acid	Muscle	**3.59E−02**	2.02E−01	9.37E−01	↓
Pentadecanoate (C15)	C16537	Fatty acid	Muscle	**2.90E−03**	**5.46E−14**	9.74E−01	↑↑
Phenylalanine	C00079	Amino acid	Muscle	**1.87E−04**	4.37E−01	9.74E−01	↑↑↑
S-Adenosylmethionine (SAM)	C00019	5'-deoxyribonucleoside	Muscle	**2.27E−03**	**3.25E−02**	9.37E−01	↑↑
Threonine	C00188	Amino acid	Muscle	**1.59E−05**	1.59E−01	9.37E−01	↑↑↑
Threonine	C00188	Amino acid	Haemolymph	**1.18E−04**	**3.11E−03**	9.83E−01	↑↑↑
Tryptophan	C00806	Amino acid	Muscle	**7.36E−03**	**3.29E−03**	9.37E−01	↓
Tryptophan	C00806	Amino acid	Haemolymph	**1.91E−02**	6.00E−01	9.83E−01	↓
Tyrosine	C00082	Amino acid	Muscle	**1.13E−03**	3.09E−01	9.37E−01	↑↑
Valine	C00183	Amino acid	Muscle	**1.94E−05**	2.05E−01	9.37E−01	↑↑↑

**Table 2:** Metabolite levels significantly different in juvenile abalone compared to adults

Metabolite	KEGG Compound ID	Classification	Tissue	Site (*p* < 0.05)	Stage (*p* < 0.05)	Interaction (*p* < 0.05)	Response Juveniles
11-Eicosenoate	C16526	Fatty acid	Muscle	6.17E−02	**2.34E−02**	9.37E−01	↑
2-Aminobutyrate	C01234	Carboxylic acid	Muscle	4.39E−01	**4.51E−02**	9.37E−01	↑
2-Oxoglutarate	C00026	Keto acid	Muscle	**1.94E−05**	**6.82E−04**	9.37E−01	↓↓
Alanine	C00133	Amino acid	Haemolymph	9.99E−01	**2.43E−07**	9.83E−01	↓↓↓
Aminomalonate	C00872	Amino acid	Muscle	5.82E−01	**6.03E−08**	9.37E−01	↑↑↑
Arachidonate	C00219	Fatty acid	Muscle	1.87E−01	**5.24E−05**	9.37E−01	↓↓↓
cis-Aconitate	C00417	Organic acid	Muscle	7.16E−01	**3.79E−07**	9.37E−01	↑↑↑
Citrate	C00158	Dicarboxylic acid	Muscle	9.59E−01	**8.47E−07**	9.37E−01	↑↑↑
Creatinine	C00791	Alpha amino acid	Haemolymph	1.12E−01	**1.78E−02**	9.83E−01	↓
Cystathionine	C02291	Amino acid	Muscle	**7.31E−05**	**5.20E−05**	1.55E−01	↓↓↓
Dihomo-gamma-linolenate	C03242	Fatty acid	Muscle	**2.63E−03**	**4.32E−03**	9.37E−01	↑↑
DL-3-Aminoisobutyrate	C03665	Carboxylic acid	Muscle	9.75E−01	**4.51E−02**	9.37E−01	↑
Dodecanoate	C02679	Fatty acid	Muscle	7.02E−02	**2.98E−02**	9.37E−01	↓
GABA	C00334	Carboxylic acid	Haemolymph	9.36E−01	**6.81E−03**	9.83E−01	↓
gamma-Aminobutyric acid (GABA)	C00334	Carboxylic acid	Muscle	9.75E−01	**2.95E−06**	1.55E−01	↓↓↓
gamma-Linolenate	C06426	Fatty acid	Muscle	2.02E−01	**1.99E−06**	9.37E−01	↑↑↑
Glutamine	C00064	Amino acid	Muscle	**1.58E−02**	**6.66E−04**	9.74E−01	↓↓
Glutamine	C00064	Amino acid	Haemolymph	9.08E−01	**9.48E−04**	9.83E−01	↓↓
Glycine	C00037	Amino acid	Muscle	9.75E−01	**3.79E−07**	9.37E−01	↑↑↑
Hexadecanoate (C16)	C00249	Fatty acids	Muscle	5.21E−02	**1.50E−02**	9.98E−01	↑
Isocitrate	C00311	Organic acid	Haemolymph	9.41E−01	**2.40E−03**	4.10E−01	↑↑
Isoleucine	C00407	Amino acid	Haemolymph	2.10E−01	**3.91E−02**	9.83E−01	↓
Isoleucine	C00407	Amino acid	Muscle	**1.59E−05**	**3.97E−02**	9.37E−01	↓
Itaconate	C00490	Carboxylic acid	Muscle	8.70E−01	**4.89E−05**	9.37E−01	↑↑↑
Itaconate	C00490	Carboxylic acid	Haemolymph	2.10E−01	**9.75E−03**	8.53E−01	↑
Lactate	C00186	Hydroxy acid	Haemolymph	9.00E−01	**1.27E−04**	8.53E−01	↓↓↓
Leucine	C00123	Amino acid	Muscle	5.82E−01	**2.06E−05**	3.19E−01	↓↓↓
Leucine	C00123	Amino acid	Haemolymph	9.08E−01	**6.63E−03**	9.83E−01	↓
Maleate	C01384	Carboxylic acid	Muscle	6.97E−02	**1.60E−04**	9.37E−01	↑↑↑
Methionine	C00073	Amino acid	Haemolymph	3.06E−01	**2.01E−02**	9.83E−01	↓
Myristoleate	C08322	Fatty acid	Muscle	**2.80E−02**	**5.18E−03**	9.37E−01	↑
NADP_NADPH	C00006	Nucleoside	Muscle	5.21E−02	**1.39E−02**	9.37E−01	↑
Ornithine	C00077	Amino acid	Muscle	**2.90E−03**	**4.89E−05**	9.37E−01	↓↓↓
Ornithine	C00077	Amino acid	Haemolymph	6.23E−01	**1.27E−04**	9.83E−01	↓↓↓
Pentadecanoate (C15)	C16537	Fatty acid	Muscle	**2.90E−03**	**5.46E−14**	9.74E−01	↑↑↑
Pentadecanoate (C15)	C16537	Fatty acid	Haemolymph	5.97E−01	**1.21E−04**	9.83E−01	↑↑↑
S-Adenosylmethionine (SAM)	C00019	5'-deoxyribonucleoside	Haemolymph	4.05E−01	**1.12E−03**	9.83E−01	↓↓
S-Adenosylmethionine (SAM)	C00019	5'-deoxyribonucleoside	Muscle	**2.27E−03**	**3.25E−02**	9.37E−01	↓
Serine	C00065	Amino acid	Haemolymph	9.00E−01	**1.38E−02**	9.83E−01	↓
Strombine	C03790	Amino acid derivative	Muscle	3.54E−01	**2.29E−03**	9.37E−01	↑↑
Succinate	C00042	Dicarboxylic acid	Haemolymph	9.41E−01	**1.85E−02**	9.83E−01	↓
Threonine	C00188	Amino acid	Haemolymph	**1.18E−04**	**3.11E−03**	9.83E−01	↓↓
Tryptophan	C00806	Amino acid	Muscle	**7.36E−03**	**3.29E−03**	9.37E−01	↑↑
Tyrosine	C00082	Amino acid	Haemolymph	9.36E−01	**4.80E−02**	9.83E−01	↑
Valine	C00183	Amino acid	Haemolymph	1.97E−01	**3.70E−02**	9.83E−01	↓

Compounds are listed in an alphabetical order, followed by KEGG identification numbers, metabolite class grouping, tissue in which the finding was made, *p*-values, and changes between the groups. Changes are grouped as higher (↑) or lower (↓) metabolite abundances based on *p*-values with < 0.05 = mild (↑); < 0.005 = moderate (↑↑) and < 0.0005 = severe (↑↑↑) changes

### Central carbon metabolism is mostly conserved

Based on video footage collected from the sampling sites, Durham was more sheltered, with less exposure to wave action, while Wharekauri was more exposed. Not only does this influence immediate nutrient and waste levels but also oxygen which could influence energy metabolism considerably. In a previous study on *H. iris*, abalone from wave exposed and sheltered sites showed large differences in the glycolytic pyruvate reductase enzyme, tauropine dehydrogenase, and glycogen content (Wells et al., 1998), hinting to perturbed carbohydrate metabolism. In this study, the concentration of several metabolites was higher in the animals from the more exposed area (Wharekauri), however, intermediates of the central carbon metabolism [glycolysis pathway and tricarboxylic acid (TCA) cycle—illustrated in Fig. 2] reflected few changes which suggests unperturbed energy production from carbohydrates. This suggests that sufficient algal sources were obtained at both sites for metabolic functioning, as previously seen in *H. laevigata* (Bansemer et al., 2016).

Central carbon metabolism results from the current study in Fig. 2, show prominent differences between adult and juvenile abalone. It can be hypothesised that juvenile abalone are in a steady growth phase, with anabolic reactions considerably upregulated. Once cell proliferation takes priority, as in the case of growing juveniles, metabolic demands change, enabling dietary nutrients to support cell growth, and manage redox challenges associated with anabolic processes. Typically, during anabolic processes, metabolites are diverted from the central carbon metabolism, to be used in the production of nucleotides, amino acids and fatty acids (Zhu & Thompson, 2019). Most of these anabolic pathways have a high energy demand which necessitates upregulation of the central carbon metabolism and oxidative phosphorylation. The TCA cycle in particular keeps functioning due to anaplerotic reactions involving branched chained amino acids (leucine, isoleucine and valine), glutamine, alanine, lactate and tyrosine (all affected in this study), which replenishes the TCA cycle to ensure production of essential energy (Inigo et al., 2021). Interestingly, in the current study, the metabolites supporting anaplerotic reactions were mostly lower in juvenile abalone, supporting the idea that these metabolites are utilised by the TCA cycle for energy purposes, as well as other pathways discussed below.

Focusing on Fig. 2, intermediates in first half of the TCA cycle (citrate, aconitate, itaconate, and isocitrate) were significantly elevated in juvenile abalone (both sites) compared to adults. 2-Ketoglutaric acid and (systemic) succinate were significantly lower in the juvenile animals. An increase in citrate for example serves as an important signal for the inhibition of catabolic reactions, favouring anabolic reactions (Frezza, 2017), supporting the hypothesis that juvenile abalone are diverting energy towards growth in the current study. Lower succinate levels could be an indication of an upregulated aspartate-succinate pathway important in the synthesis of nicotinamide adenine dinucleotide (NAD^+^) and flavin adenine dinucleotide (FAD) to support energy production in the presence of stressors (Venter et al., 2018a).

### Lipogenesis in Wharekauri abalone apparently upregulated

The concentrations of several saturated and unsaturated fatty acids, illustrated in Fig. 3, were elevated in the muscle of abalone collected at the Wharekauri site (both adults and juveniles). Since these animals are considered faster growers, it is possible that fatty acid synthesis from glucose via de novo lipogenesis is upregulated in these animals, as cells accumulate lipids under conditions such as growth (Giese, 1966). Additionally, the diet consumed by the abalone at Wharekauri can serve as a rich source of fatty acids (lipids), attributing to the higher levels of muscle 9E-heptadecenoate, dihomo-gamma-linolenate, DPA, myristate, myristoleate and pentadecanoate. Members of Bacillariophyceae (diatoms) are abundant in aquatic habitats and are considered as the most important primary producers of fatty acids in marine food chains (Zhukova, 2019). Similar outcomes have been reported where the fatty acid composition of lipid samples reflected those of the diets consumed by the animals, as seen in *H. asinia* (Bautista-Teruel et al., 2011), *H. discus hannai* Ino (Xu et al., 2004) and *H. tuberculate* (Hernández et al., 2013).

Malonate and gamma-linolenate were significantly lower in the haemolymph of Wharekauri abalone (Fig. 3). Malonate is typically used as a precursor for *de nov*o synthesis of fatty acids (Guan & Nikolau, 2016), but can also act as a competitive inhibitor of succinate dehydrogenase, limiting mitochondrial respiration (Bowman & Wolfgang, 2019). In addition, gamma-linolenate plays a role as an polyunsaturated fatty acid, vital towards the production of eicosanoids (signalling molecules) (Sergeant et al., 2016). From this context, these haemolymph metabolites support the utilisation thereof by muscle metabolites, where fatty acids are incorporated into cells via a process that is not energy dependent but requires additional proteins (amino acids), which does require energy for fatty acid transport (Laposata, 1995).

The concentration of most fatty acids differed markedly between the adult and juvenile abalone (seen in Fig. 3). Increased citrate levels are associated with elevated lipogenesis (Lee et al., 2018), which correspond with the elevated long chain fatty acids detected in the muscle tissue of juvenile animals. Upregulated lipogenesis and storage of dietary lipids not only ensure a supply of triacylglycerols for later use, but also phospholipids needed for cell division (Schönfeld & Wojtczak, 2016; Tang et al., 2018). Juvenile, *H. asinia,* showed weight gains following feeding experiments with supplemented essential fatty acids (Bautista-Teruel et al., 2011), while research on juvenile *H. discus hannai*, supported the accumulation of fatty acids in soft tissue, due to algal diets (Pan et al., 2018). This study supports the function of fatty acids in juvenile *H. iris,* as an anabolic source, that assists with organismal growth. Additionally, lower levels of dodecanoate (a medium chain fatty acid) were found, suggestive of down-regulation of long chain fatty acids.

### Wharekauri abalone retains amino acids (and nitrogen) better

The concentrations of most amino acids and many of their associated intermediates were elevated in the muscle of Wharekauri abalone compared to the Durham animals (Fig. 4). Higher concentrations of amino acids can be linked to anabolic activities, promoting growth, as previously reported in *H. midae* (Venter et al., 2018c). Generally amino acids are degraded by different pathways that feed into energy production pathways, yet in scenarios where sufficient carbohydrate sources are present for energy production, amino acids can fulfil other functions like osmoregulation (Venter et al., 2018a), production of proteins and neuropeptides (Sharker et al., 2020) and shell formation (Marie et al., 2010). When excessive glycolytic activity is experienced, associated intermediates are diverted to other pathways assisting with the production of non-essential amino acids and lipids, typically required for cell growth (Zhu & Thompson, 2019).

Apart from upregulated protein synthesis, the carbon and nitrogen derived from these affected amino acids can also support nucleotide production. Elevated methionine and S-adenosylmethionine (SAM) concentrations could also be linked to down-regulated gene methylation (Roznere et al., 2017), which could contribute to the faster growth rate in the Wharekauri abalone. Lower concentrations of serine, threonine, SAM and methionine were detected in juvenile haemolymph collected at both sites (Fig. 4). Since these metabolites are used by the folate- and methionine cycle for the regulation of gene expression, energy balance and the synthesis of biomacromolecules (i.e., nucleotides) (Pan et al., 2021), it could lead to the same conclusion that gene silencing is possibly down-regulated.

As seen in Fig. 4, the branched chain amino acids were markedly elevated in the muscle of the Wharekauri abalone (especially the adults) which highlight their ability to retain essential amino acids for anabolic processes or as alternative fuel to replenish TCA cycle intermediates when in catabolic state. Likewise, the aromatic amino acids (phenylalanine and tyrosine) with their associated intermediates were also elevated in the Wharekauri group, as seen in fast growing *H. midae* (Venter et al., 2018c). Additional amino acids and metabolites affected in this study can be attributed towards an antioxidant function. A study on *H. midae* reported that juvenile abalone are more resistant to oxidative stress because they have higher levels of antioxidant enzymes (Vosloo et al., 2013). Juvenile *H. iris*, from this study, displayed lower concentrations of metabolites relating to the glutathione metabolic pathway (glutamine from glutamate, and ornithine) and oxidative stress mechanisms (serine, cystathionine, methionine and GABA), showcasing that these animals had no requirement to scavenge reactive oxygen species or respond metabolically to stressors (Delorme et al., 2021). Indeed, urea cycle-related intermediates were also lower in juvenile abalone in both sites which highlights the retention of nitrogen. The urea cycle also functions as a collection point for nitrogenous waste (Azizan et al., 2021), especially during stressed conditions as previously demonstrated in abalone (Venter et al., 2018b).

Glycine, one of the severely elevated metabolites detected in the juveniles (Fig. 4), contributes to the biosynthesis of heme, purines, creatine, glutathione and uric acid, which would require upregulated de novo glycine synthesis (Venter et al., 2018c). Furthermore, during anaerobic conditions glycine is utilised to produce strombine, releasing NAD^+^ for use in the glycolysis pathway. Higher strombine was also found in the current study, which links to the increased glycine levels. The production of strombine can be considered as a valuable carbon chain store (like pyruvate and glycine) for use in the abovementioned pathways and TCA cycle. It is arguably also a better storage form than lactate, with better buffer capacity (Venter et al., 2018a). In contrast to strombine, lactate along with alanine, were significantly lower in the haemolymph samples from juvenile abalone tested. Lactate is a well-known metabolite of anaerobic metabolism, often reported as increased in abalone subjected to stress (Alfaro et al., 2021). Elevations in alanine have also been attributed to abalone as a mechanism to buffer H^+^ ions during stress and to regulate intracellular osmotic pressure. Moreover, the reduction hereof in juvenile abalone reflects the transport function of haemolymph where metabolites central to the glycolysis pathway and TCA cycle are absorbed from the haemolymph by tissues (muscle) where they can be utilised (Venter et al., 2018b).

### Fishery considerations

This research provides unique physiological insights towards abalone populations of New Zealand and globally. In the Chatham Islands, Wharekauri is classified as a high abalone catch area, where animals typically achieve the required fishing size in a shorter time period, arguably due to their surrounding conditions and the interaction with their environment. A similar trend was reported in greenlip abalone, where better quality habitat positively influenced growth patterns (Dixon & Day, 2004). Abalone populations are classified as stunted (slow growers), where individuals grow slowly and/or achieve a smaller maximum size in comparison with other populations, and has been observed in greenlip abalone (*H. laevigate*) (Hart et al., 2013), and black-footed abalone (*H. iris*) (Naylor & Andrew, 2004). Additionally, protected marine areas are being sought out to aid the restoration of white, pink (*H. corrugate*) and green abalone (*H. fulgens*) (Rogers‐Bennett et al., 2002). This brings to light the need to characterise “fast-growing/non-stunted” areas to provide biomarkers relating to the biological response of abalone to their environment, for potential reseeding or translocating initiatives. The current research provides insight into Chatham Islands abalone sites, where the translocation of abalone from other populations to Wharekauri can be achieved, as long as abalone metabolic energy is invested into anabolic activities and not maintenance or recovering from stress, resulting in limited energy for growth (Venter et al., 2018c). Freshwater mussel research proved that characterisation of metabolite levels can provide a framework for management decisions and the development of tools to improve conservation techniques (Roznere et al., 2017). Yet, various environmental variables will also need to be considered, as demonstrated in caged mussels, where the effect of seasonal patterns on condition and tissue biochemistry have been highlighted as other considerations for translocation (Gray & Kreeger, 2014), something which is unknown for *H. iris* at this stage. Additional research on *H. iris*, identified faster growing animals in areas were the mean monthly maximum sea surface was lower, while the opposite was also true, where slow growth was linked with higher temperatures (Naylor et al., 2006). From the current investigation no temperature data was collected, creating an important factor to take into account in future studies and translocation strategies.

Metabolomics results of juvenile abalone indicated that anabolic metabolism is dominant, supporting steady growth mechanisms, suggesting that juvenile abalone can be translocated around the Chatham Islands. In saying that, biomarker analyses will be essential to monitor animal physiology and the metabolic response to environmental conditions. For example, the survival of juvenile *H. roei* was compromised by high wave energy and temperature environment (Strain et al., 2019). Restoration work on juvenile *H. kamtschatkana* revealed that monitoring of genetic diversity of hatchery-produced and habitat quality are required to ensure survival (Read et al., 2012). The use of metabolomics to identify candidate biomarkers in abalone following exposure to stressors have been well-documented, yet validation of these candidate biomarkers remains largely unexplored (Nguyen et al., 2021).

## Conclusions

From the metabolomics results, it is clear that fast growing abalone from Wharekauri obtained sufficient carbohydrate sources from the environment to support energy production functions. Additionally, the metabolite profiles showed increased fatty acid synthesis essential for structural functions and higher amino acids to support protein synthesis. In juvenile abalone, energy produced via glycolysis and the TCA cycle was utilised to support anabolic reactions and the production of amino acids and fatty acids, corresponding to nucleotide production for cell growth. The lack of increased metabolites previously associated with abalone subjected to stress infers the idea that juveniles in this study were investing energy in growth as opposed to spending energy on maintenance or recovering processes. This study showcases metabolomics as a valuable tool for investigating the altered metabolic processes related to growth in abalone, and hence, is a valuable tool for identifying biomarkers for growth and health monitoring to support a growing and more sustainably abalone fishery.

## Supplementary Information

Below is the link to the electronic supplementary material.

Supplementary file1 (DOCX 356 kb)

## Data Availability

Data supporting the findings of this study can be obtained from the authors upon reasonable request.
