# Supplementary material for: Metabolite profiling of abalone (Haliotis iris) energy metabolism: a Chatham Islands case study
Source: Metabolomics. 2022 Jul 13;18(7):52. doi: 10.1007/s11306-022-01907-6 (PMC9279229; doi:10.1007/s11306-022-01907-6)
Supplement: Supplementary file 1 — Supplementary file1 (DOCX 356 kb) [file 11306_2022_1907_MOESM1_ESM.docx]

## Supplementary material

### S1. Metabolomics analysis

Frozen haemolymph (500 µL) together with 20 µL of internal standard (10 mM L-alanine-2,3,3,3-d_4_), were dried for 4 h at 0°C on a SpeedVac concentrator with a refrigerated vapor trap. Muscle samples were ground using a mortar and pestle following freeze drying of the collected subsample. Approximately 10 mg of tissue sample were weighed and used for metabolite extraction with 20 µL of internal standard. A two-step sequential extraction method was used for metabolite extraction as described previously (Nguyen et al., 2018). In brief, 500 µL of a cold 1:1 methanol:water solution was added to the dried samples, vortexed for 1 min and centrifuged for 10 min at 20 800*g* at -4ºC. The process was repeated using 500 µL of a cold 4:1 methanol:water solution; whereafter the supernatants were combined and dried using a SpeedVac.

Extracted metabolites were derivatised by methyl chloroformate (MCF) alkylation using an established protocol (Smart et al., 2010). Dried samples were re-suspended in 400 µL 1M sodium hydroxide and quantitatively transferred to salinized borosilicated glass tubes, followed by the addition of 334 µL methanol and 68 µL pyridine. This was followed by a series of reagent additions and vortexing: 40 μL of MCF reagent - 30s, 40 μL of MCF - 30s, 400 μL of chloroform - 10s, and 800 μL of 50 mM sodium bicarbonate – 10s. The mixture was centrifuged at 1174*g* for 5 min at 6°C. The upper aqueous layer was discarded, and a small amount of anhydrous sodium sulphate was added to remove residual water. The chloroform phase containing the MCF derivatives was transferred to 2-mL amber GC glass vials fitted with inserts for GC-MS analyses.

Quality control (QC) samples were included in every batch by preparing a pooled mixture of haemolymph or muscle tissue samples. These QC samples were included within the biological sample batches and prepared by taking a small volume of each biological sample, thoroughly mixed into a homogenous pooled sample (Broadhurst et al., 2018), and treating them as experimental samples. The QC samples were injected at regular intervals throughout the analytical run of the analysed batch to measure repeatability and to identify any potential batch effects in the data. Additionally, a derivatised sample blank containing the internal standard, an in-house prepared derivatised standard amino acid mix, a non-derivatised standard alkane mix (Supelco 49451-U, Merck), and a sample of pure chloroform solvent were also injected and analysed for QC purposes (Young et al., 2019).

The MCF derivatives were analysed with an Agilent GC7890B and autosampler coupled to a MSD5977A (Agilent Technologies), with a quadrupole mass selective detector (EI) operated at 70 eV. The system was equipped with a ZB-1701 GC capillary column (30m×250 μm id × 0.15 μm with 5m stationary phase). Helium was used as the carrier gas (flow of 1 mL min^−1^) and the injector temperature was heated to 260°C. Samples (1 μL) were injected under pulsed splitless mode with the injector temperature at 260°C. The helium gas flow through the GC-column was set at a constant flow of 1 mL min^−1^. The GC-oven temperature was initially held at 45°C for 2 min, and then raised with a gradient of 9°C min^−1^ to 180°C. After 5 min, the temperature was increased at 40°C min^−1^ to 220°C. After a further 5 min, the temperature was increased at 40°C min^−1^ to 240°C and held for 11.5 min. Finally, the temperature was increased at 40°C min^−1^ until it reached 280°C, where it was held for a further 2 min. The interface temperature was set to 250°C, the source was set at 230°C and the quadrupole temperature was set at 150°C. The mass spectrometer was operated in scan mode (starting after 6 min solvent delay; mass range 38–650 amu at 1.47 scans sec^−1^). Identification of compounds was carried out using mass spectra acquired in scan mode from 38 to 550 amu, with detection threshold of 100 ion counts (Smart et al., 2010).

### Data processing

Raw spectra were processed using Automated Mass Spectral Deconvolution and Identification System (AMDIS v2.66) software. Metabolite identifications and peak integrations (relative quantification) were conducted using Chemstation Software (Agilent Technologies) and customised R-XCMS based scripts (Aggio et al., 2011), based on an in-house mass spectral library of MCF derivatised commercial standards. Compound identifications were based on matches (≥ 70%) to both the MS spectrum of the derivatised metabolite and its respective chromatographic retention times. Identified compounds can be assigned a level 1 identification confidence level (Schymanski et al., 2014). Unknown features are not shown. Data were blank-corrected and aberrant records were removed. The data matrices of peak intensities were pre-processed for quality control purposes and to meet the distributional requirements prior to statistical analyses using the web-based tool MetaboAnalyst 5.0 (Pang et al., 2021). Data were normalised to sample biomass in the case of muscle tissue, and to the peak intensity of the internal standard (d_4_-alanine) (Nguyen et al., 2018).

### S2. PCA loadings of Site differences

The contribution of the metabolites in the clustering of the site-specific PCA scores is shown in the following table and accompanying loadings plot. The samples (scores) of the different sites showed moderate separation on PC1, hence the coefficients in Table S1 is sorted accordingly.

**Table S1: Variable coefficients of the first three PCs (explaining most of the site variance)**

|  | PC1 | PC2 | PC3 |
| --- | --- | --- | --- |
| gamma-Linolenate | -0.14595 | -0.14696 | -0.33814 |
| (11E)-Octadecenoate | -0.1437 | -0.10281 | -0.35741 |
| Malonate | -0.06386 | -0.03039 | -0.07351 |
| 2-Oxoglutarate | -0.03877 | -0.10753 | 0.016552 |
| Tryptophan | -0.03696 | 0.081057 | 0.011295 |
| (9E)-Hexadecenoate (C16:1n9) | -0.01316 | 0.038862 | -0.03584 |
| Pentadecanoate (C15) | 0.009832 | 0.58145 | -0.08071 |
| Myristoleate | 0.031923 | 0.12913 | 0.037785 |
| Glutamate | 0.065537 | 0.013667 | 0.072519 |
| Myristate (C14) | 0.068027 | 0.085465 | 0.019162 |
| Docosapentaenoate (DPA) | 0.079251 | 0.14034 | -0.06821 |
| 9E-Heptadecenoate | 0.092398 | 0.11242 | -0.00949 |
| Dihomo-gamma-linolenate | 0.095609 | 0.3022 | -0.03401 |
| Lysine | 0.10835 | 0.11136 | 0.095066 |
| Tyrosine | 0.1084 | 0.1128 | 0.064059 |
| Methionine | 0.11174 | 0.013977 | 0.011776 |
| Phenylalanine | 0.11615 | 0.026388 | 0.05873 |
| Creatinine | 0.11872 | 0.005111 | 0.1461 |
| beta-Alanine | 0.12246 | 0.16618 | 0.045351 |
| Cysteine | 0.12365 | 0.046908 | 0.051177 |
| Valine | 0.12667 | 0.020526 | 0.09547 |
| cis-Aconitate | 0.13037 | 0.25197 | -0.11128 |
| Isoleucine | 0.13394 | -0.00448 | 0.081139 |
| Aminoadipate | 0.13535 | 0.032972 | 0.041659 |
| S-Adenosylmethionine | 0.15052 | -0.07902 | 0.051016 |
| Ornithine | 0.15421 | -0.15041 | 0.070612 |
| Histidine | 0.16324 | 0.092214 | 0.15985 |
| 1-Aminocyclopropane-1-carboxylate (ACC) | 0.20129 | -0.00683 | 0.11331 |
| 4-Hydroxyphenylacetate | 0.20563 | 0.27333 | 0.27437 |
| Glutamine | 0.21474 | -0.2057 | 0.069413 |
| O-Acetylserine | 0.3222 | -0.0019 | -0.21654 |
| Cystathionine | 0.42321 | -0.43103 | 0.23716 |
| Threonine | 0.51851 | -0.01612 | -0.65543 |


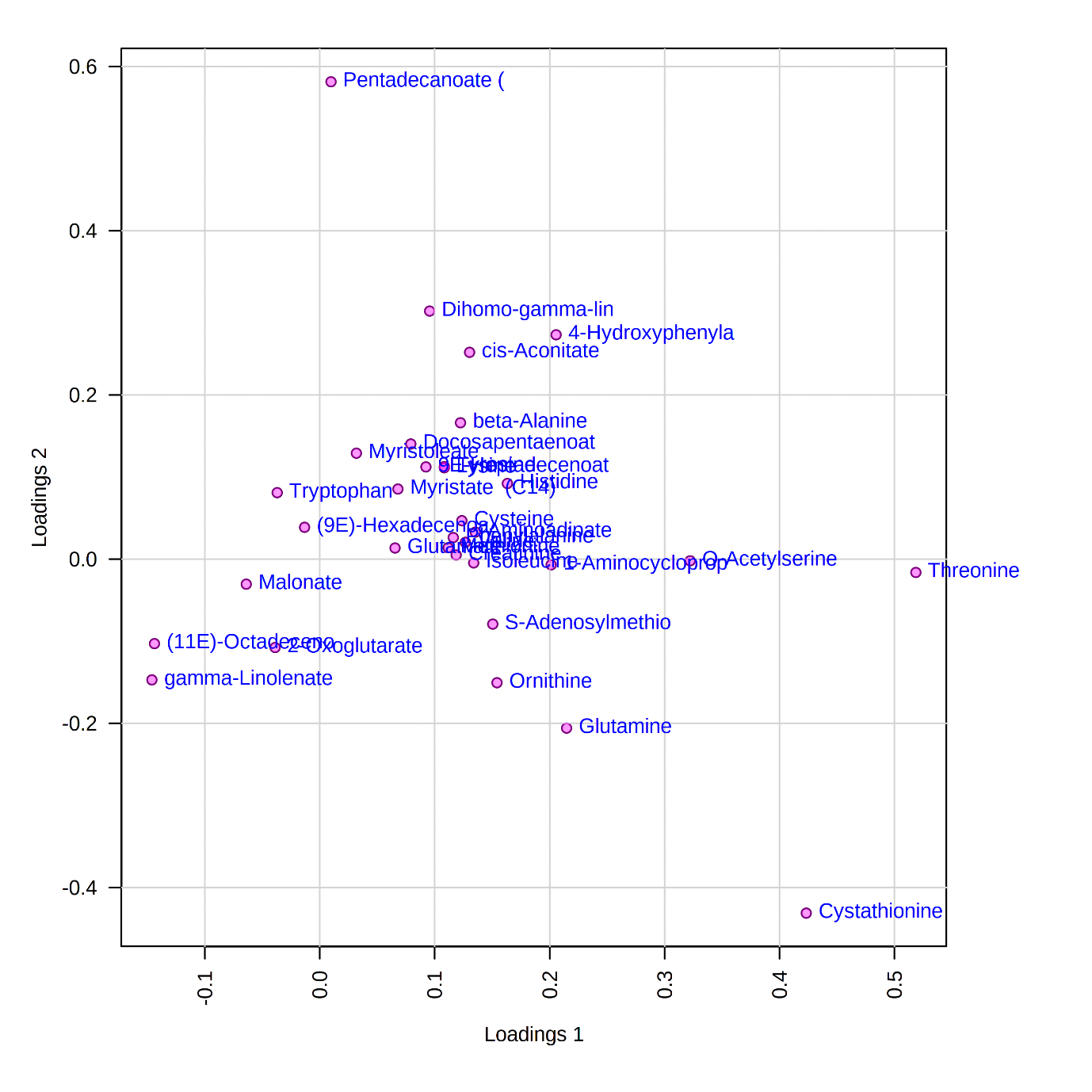


**Figure S1:** Loadings plot showing the contribution of metabolites to the distribution of scores (and grouping of samples from similar site).

### S3. PCA loadings of Stage differences

The contribution of the metabolites in the clustering of the site-specific PCA scores is shown in the following table and accompanying loadings plot. The samples (scores) of the different sites showed moderate separation on PC1, hence the coefficients in Table S2 is sorted accordingly.

**Table S2: Variable coefficients of the first three PCs (explaining most of the stage variance)**

|  | PC1 | PC2 | PC3 |
| --- | --- | --- | --- |
| Pentadecanoate (C15) | -0.22931 | 0.13621 | 0.23517 |
| Aminomalonate | -0.21353 | 0.11004 | 0.17389 |
| Strombine | -0.17332 | 0.062687 | 0.20277 |
| cis-Aconitate | -0.1485 | 0.038333 | 0.16844 |
| Glycine | -0.12247 | 0.057171 | 0.13927 |
| Citrate | -0.11481 | 0.084922 | 0.15033 |
| gamma-Linolenate | -0.10969 | 0.069974 | 0.14303 |
| Itaconate | -0.08796 | 0.045578 | 0.14426 |
| Maleate | -0.08742 | 0.047798 | 0.14491 |
| Arachidonate | -0.08522 | 0.036017 | 0.17097 |
| Tryptophan | -0.07696 | 0.050898 | 0.05273 |
| NADP_NADPH | -0.07517 | 0.041848 | 0.2248 |
| Isocitrate | -0.07398 | 0.23825 | -0.01223 |
| Dihomo-gamma-linolenate | -0.06064 | 0.090264 | 0.27204 |
| 2-Aminobutyrate | -0.06017 | 0.006179 | 0.070646 |
| Itaconate.1 | -0.0593 | 0.11631 | 0.06409 |
| 11-Eicosenoate | -0.04366 | 0.029389 | 0.13661 |
| Myristoleate | -0.04041 | 0.011326 | 0.10744 |
| Hexadecanoate (C16) | -0.03756 | 0.010183 | 0.10429 |
| DL-3-Aminoisobutyrate | -0.03611 | 0.013822 | 0.023962 |
| Tyrosine | -0.01061 | 0.56899 | 0.21566 |
| 2-Oxoglutarate | 0.032322 | 0.000666 | -0.06623 |
| Isoleucine | 0.039905 | -0.0679 | 0.1626 |
| S-Adenosylmethionine | 0.052067 | -0.09799 | 0.16946 |
| Dodecanoate | 0.056275 | -0.01333 | -0.02894 |
| Leucine | 0.072073 | -0.06013 | 0.045624 |
| gamma-Aminobutyric acid (GABA) | 0.078755 | -0.05868 | 0.013447 |
| Glutamine | 0.10156 | -0.19115 | 0.2088 |
| Valine | 0.12412 | 0.14294 | 0.013781 |
| Isoleucine.1 | 0.12919 | 0.15066 | 0.021855 |
| Creatinine | 0.13252 | 0.13622 | -0.0028 |
| Serine | 0.14429 | 0.12446 | 0.092304 |
| Alanine | 0.15248 | -0.00545 | 0.040811 |
| GABA | 0.1652 | 0.12671 | 0.059013 |
| Leucine.1 | 0.16603 | 0.1254 | 0.066669 |
| Cystathionine | 0.18342 | -0.41632 | 0.53568 |
| Methionine | 0.18486 | 0.16886 | 0.019963 |
| S-Adenosylmethionine.1 | 0.18815 | 0.14451 | -0.02123 |
| Glutamine.1 | 0.20139 | 0.11458 | 0.052924 |
| Threonine | 0.25768 | 0.27235 | -0.11042 |
| Ornithine | 0.28305 | 0.14935 | 0.057533 |
| Succinate | 0.37128 | 0.068658 | 0.1788 |
| Lactate | 0.38171 | -0.17612 | 0.15374 |


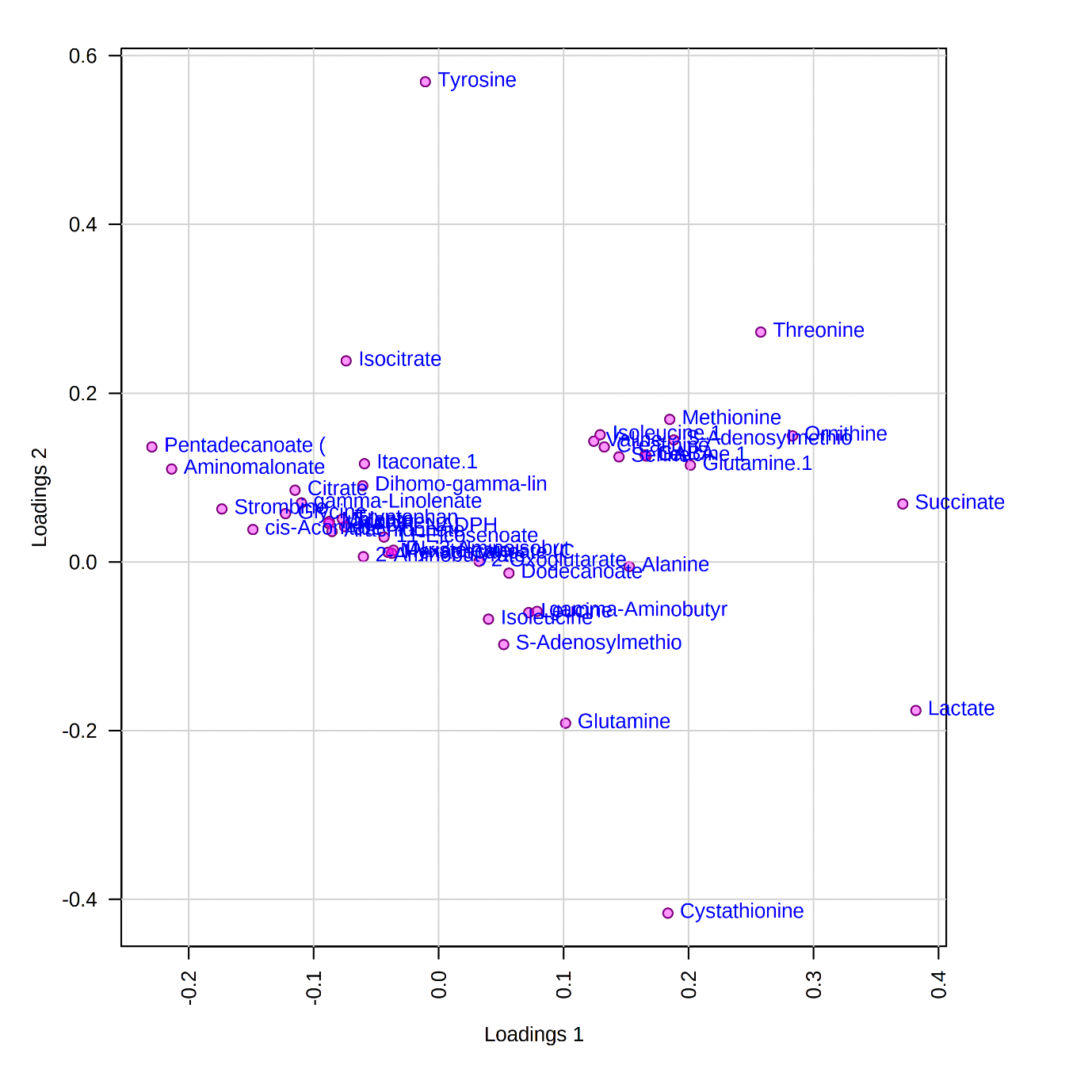


**Figure S2:** Loadings plot showing the contribution of metabolites to the distribution of scores (and grouping of samples from similar stages).

### References

AGGIO, R., VILLAS− BÔAS, S. G. & RUGGIERO, K. 2011. Metab: an R package for high-throughput analysis of metabolomics data generated by GC-MS. *Bioinformatics,* 27**,** 2316-2318.

BROADHURST, D., GOODACRE, R., REINKE, S. N., KULIGOWSKI, J., WILSON, I. D., LEWIS, M. R. & DUNN, W. B. 2018. Guidelines and considerations for the use of system suitability and quality control samples in mass spectrometry assays applied in untargeted clinical metabolomic studies. *Metabolomics,* 14**,** 1-17.

NGUYEN, T. V., ALFARO, A. C., YOUNG, T., RAVI, S. & MERIEN, F. 2018. Metabolomics study of immune responses of New Zealand greenshell™ mussels (*Perna canaliculus*) infected with pathogenic *Vibrio sp*. *Marine Biotechnology,* 20**,** 396-409.

PANG, Z., CHONG, J., ZHOU, G., DE LIMA MORAIS, D. A., CHANG, L., BARRETTE, M., GAUTHIER, C., JACQUES, P.-É., LI, S. & XIA, J. 2021. MetaboAnalyst 5.0: narrowing the gap between raw spectra and functional insights. *Nucleic acids research,* 49**,** W388–W396.

SCHYMANSKI, E. L., JEON, J., GULDE, R., FENNER, K., RUFF, M., SINGER, H. P. & HOLLENDER, J. 2014. Identifying small molecules via high resolution mass spectrometry: communicating confidence. *Environmental Science & Technology,* 48**,** 2097–2098.

SMART, K. F., AGGIO, R. B., VAN HOUTTE, J. R. & VILLAS-BÔAS, S. G. 2010. Analytical platform for metabolome analysis of microbial cells using methyl chloroformate derivatization followed by gas chromatography–mass spectrometry. *Nature protocols,* 5**,** 1709.

YOUNG, T., WALKER, S. P., ALFARO, A. C., FLETCHER, L. M., MURRAY, J. S., LULIJWA, R. & SYMONDS, J. 2019. Impact of acute handling stress, anaesthesia, and euthanasia on fish plasma biochemistry: implications for veterinary screening and metabolomic sampling. *Fish physiology and biochemistry,* 45**,** 1485-1494.
